# Frontlines of Climate Change and Global Health Inequity: How Recurring Cyclones Undermine Health, Livelihoods, and Development in the Indian Sundarbans

**DOI:** 10.5334/aogh.5074

**Published:** 2026-04-01

**Authors:** Pranay Narang, Monalisha Sahu, Monalisa Datta, Nilanjana Ghosh, Shilpa Mondal, Indrani Bhattacharya, Gaurab Basu

**Affiliations:** 1 University of California, Berkeley / UCSF Joint Medical Program, Berkeley, California, United States; 2 Philip R. Lee Institute for Health Policy Studies, University of California, San Francisco, San Francisco, California, USA; 3 All India Institute of Hygiene and Public Health, Kolkata, West Bengal, India; 4 Child In Need Institute, Kolkata, West Bengal, India; 5 Brigham and Women’s Hospital, Division of Global Health Equity, Harvard Medical School, Harvard T.H. Chan School of Public Health, Boston, Massachusetts, United States

**Keywords:** climate change, health equity, extreme weather, poverty, womens health, malnutrition

## Abstract

This study investigates the cascading impacts of recurrent cyclones on the physical and mental health, livelihoods, infrastructure, well-being, and long-term development of communities in the Indian Sundarbans, one of the world’s most climate-vulnerable regions. Semi-structured interviews were conducted with community members, frontline public health workers government officials, NGO leaders, mental health counselors, and non-licensed village doctors.

We aimed to identify the ecological, geographic, and socioeconomic conditions exacerbating the region’s vulnerability to cyclones; examine the intersecting short-term and long-term health, economic, and social impacts; characterize existing response systems; identify structural barriers impeding long-term recovery and development; and propose stakeholder-informed recommendations to strengthen disaster preparedness and promote lasting resilience and recovery.

Findings reveal that these communities remain entrapped in a recurring cycle of disaster and inadequate recovery marked by saline water intrusion, collapsed infrastructure, displacement to overcrowded shelters, loss of agricultural land, infectious outbreaks, disruptions to healthcare delivery and child education, life-threatening health emergencies, damage to livelihoods, food insecurity, and rising gender inequity, trauma, and depression. These vulnerabilities and impacts are perpetuated by chronic underinvestment and a lack of responsive policy.

Participants called for solutions such as the following: (1) pre-positioning food, water, and medicines to strengthen disaster preparedness; (2) digitizing educational certificates to mitigate school dropout; (3) expanding insurance coverage, compensation schemes, vocational training, and employment opportunities to mitigate income losses; (4) establishing mangrove reforestation programs for livelihood diversification and bolstering natural ecological defense; and (5) increasing investment in resilient infrastructure, especially hospitals, roads, and homes. Policy reforms and tax incentives could mobilize private sector investment.

The lived experiences captured in this study illuminate the daily struggle for basic security, income, health, education, and survival in the Indian Sundarbans and the urgent need for policies that uphold health equity and dignity in the face of accelerating climate threats.

## Introduction

Fossil fuel combustion and ecological degradation are disrupting our climate and planetary systems, leading to worsening heatwaves, declining food and water security, deteriorating air quality, and more frequent extreme weather events, such as cyclones, floods, and wildfires. These shifts are also altering the distribution and incidence of infectious diseases, including vector-borne illnesses. These public health threats have and will continue to result in forced migration and displacement. Together, these impacts have vast implications for physical and mental health, as well as for the social stability of communities globally [1–10].

Despite contributing minimally to greenhouse gas emissions (GHGs), impoverished communities across the Global South like those living in the Sundarbans disproportionately bear the burden of their impacts. From 1990 to 2015, the wealthiest 10% of the global population (630 million) produced 52% of emissions, while the poorest 50% (3.1 billion people) accounted for only 7% [11].

India, responsible for roughly 7% of global GHGs, is both a major emitter and among the most vulnerable nations due to its geography, large population, agricultural dependence, and climatic variability [12, 13]. One significant threat is the impact of tropical cyclones on poor coastal communities. The country’s east coast lies within the North Indian Ocean (NIO) basin, a region highly susceptible to recurring tropical cyclones [14]. While the NIO accounts for only 6% of tropical cyclones each year globally [15], this basin has produced over 75% of tropical cyclones that have caused at least 5,000 fatalities per event over the past three centuries [16]. Research has observed that increases in the frequency and probability of major tropical cyclones are consistent with modeling projections of intensity trends under global warming [17].

Within this high-risk region lies the Sundarbans, the world’s largest contiguous mangrove forest and a UNESCO World Heritage Site, spanning the border of West Bengal, India, and Bangladesh [18, 19]. This low-lying deltaic ecosystem of mudflats, mangroves, barren lands, and tidal streams lies at the frontline of cyclone devastation, flooding, monsoons, sea-level rise, coastal erosion, saline intrusion, and heatwaves [20]. Recent storms highlight the escalating severity of these threats for the 4.5 million people inhabiting the Indian Sundarbans [21]. Cyclone Fani (2019, Category 4) impacted 1 million people and cost $8.1 billion in damages; cyclone Bulbul (2019, Category 3) affected 3.5 million people and cost $3.37 billion in losses; and cyclone Amphan (2020, Category 5) disrupted 60 million people’s lives, cost $13 billion in damages, destroyed 1.5 million dwellings, and displaced 3 million people [22–28].

From a global perspective, the Sundarbans exemplifies how the climate crisis undermines cross-sectoral development and disproportionately harms those who have contributed least to its cause. Despite decades of international negotiations at the United Nations Climate Change Conference of Parties (COPs) to mobilize climate financing for emissions reduction, adaptation, and loss-and-damage, progress remains far short of the estimated need [29]. In 2024, at COP29, countries agreed to increase the current $100 billion annual financing target from wealthier nations to $300 billion by 2035 [29]. If fulfilled, this marks a significant step forward yet far below the $1 trillion per year needed by 2030 to keep the goals of the Paris Agreement within reach [30, 31].

Life in the Sundarbans is marked by poverty, deprivation, and recurring struggles against the impacts of tropical cyclones. A significant portion of households rely on subsistent cyclone-sensitive livelihoods, including farming, fishing, and honey collection, creating pervasive socioeconomic precarity and food insecurity in the wake of repeated income losses [32]. Over half the population—approximately 2.2 million people—live below the poverty line [27, 33]. Healthcare delivery remains severely underdeveloped, with acute shortages of primary health centers (PHCs) and medical personnel [34]. Moreover, the region faces persistent infrastructural challenges, including fragile roads, a shortage of cyclone shelters, and degraded embankments [33].

Within this broader context, Gosaba and Kultali, two blocks in South 24 Parganas, West Bengal, host some of the most vulnerable communities in the Sundarbans. Their exposure to recurring cyclone-driven storm surges, embankment breaches, land degradation, and pervasive crop loss is heightened by the low elevation of homes and proximity to tidal creeks for most households and infrastructure [35].

Few studies have investigated the significant, intersecting impacts of tropical cyclones on the physical and mental health, infrastructure, livelihoods, and overall well-being of coastal communities in the Sundarbans and the barriers impeding post-cyclone recovery and long-term development. Moreover, existing studies rarely interrogate these complex structural and social impacts through the lens of health equity; and no investigation has offered stakeholder-informed recommendations for community leaders and policymakers. Elevating the lived experiences of coastline communities such as the Sundarbans into global climate policy offers several benefits, including raising awareness of urgent societal needs, holding policymakers accountable to the human costs of delayed action, and compelling more accelerated, ambitious investment.

To address this gap, we interviewed frontline community members, public health workers, government officials, non-governmental organization (NGO) leaders, and health professionals to describe (1) the ecological, geographic, and socioeconomic conditions predisposing the region to repeated cyclone impacts; (2) the immediate and long-term health, economic, infrastructural, and social impacts of cyclones; (3) existing response and recovery systems and capabilities; (4) structural barriers that hinder effective response and long-term recovery; and (5) community-led solutions for strengthening disaster response and recovery.

## Methods

### Study design and recruitment

We employed a qualitative methodology using semi-structured interviews to explore participants’ perspectives, experiences, and recommendations regarding cyclone impacts, vulnerabilities, existing resources, and policy and investment gaps in accordance with the research aims. Reflexive thematic analysis, incorporating deductive and inductive approaches, was used to identify recurring themes from the data [36, 37].

Interviews were conducted with a diverse cohort of stakeholders to understand a broad spectrum of perspectives, including community members (farmers, drivers, non-licensed village doctors, shopkeepers, and women), frontline public health workers (e.g., Accredited Social Health Activists, or ASHAs; Anganwadi Workers, or AWWs); elected local government officials (Gram Panchayat Pradhan; Deputy Gram Panchayat Pradhan); appointed local government officials (public health nursing officers, block-level epidemiologist); mental health counselors; senior leadership from a locally embedded NGO; and a professor of disaster management from a flood-prone adjacent state (Table 1). Purposive and convenience sampling were employed to deliberately recruit individuals with direct experience living through and responding to cyclones. Recruitment was limited to Kultali and Gosaba, two vulnerable blocks, where the locally embedded NGO had strong community ties. These sites were also prioritized for their repeated exposure to severe cyclones.

**Table 1:** Characteristics of study participants (n = 33).

Gender	Value*
Female	20 (60.6%)
Male	13 (39.4%)
**Current Residence, n (%)**	
Kultali	7 (21.2%)
Gosaba	19 (57.6%)
Kolkata, West Bengal	7 (21.2%)
**Occupation and Role, n (%)**	
Locally Embedded NGO Leadership	5 (15.2%)
Mental Health Professionals	2 (6.1%)
Academic Professor of Disaster Management	1 (3%)
Elected Government Official	3 (9.1%)
Appointed Government Official	4 (12.12%)
Accredited Social Health Activists (ASHA Worker)**	3 (9.1%)
Anganwadi Worker (AWW)***	4 (12.12%)
**Community Members**	11 (33.33%)
Driver	2 (6.1%)
Farmer	2 (6.1%)
Shopkeeper	1 (3%)
Homemaker	2 (6.1%)
Women’s Self-Help Group (SHG) Member****	2 (6.1%)
Non-Licensed Village Doctor	2 (6.1%)

*Data are expressed as number (%), unless noted.

**Accredited Social Health Activists (ASHA Worker): female community health worker under India’s National Health Mission who serves as the critical link between rural households and health services. Selected from within the community, ASHAs support maternal and child care, facilitate sanitation and nutrition programs, assist with immunizations, promote basic health education, and contribute to health data and local planning [38].

***Anganwadi Worker (AWW): female community health worker at Integrated Child Development Service (ICDS) centers, a national program providing supplementary nutrition, preschool education, nutrition and health education, immunizations, health check-ups, and referrals to support children under six and their mothers with the goal of reducing child malnutrition, illness, and school dropout [39].

****Women’s Self-Help Group (SHG) Member: A member of a 12–18-person women-only collective that meets monthly to discuss finances, pool savings, access bank loans, and provide small loans to support other women’s needs and promote self-reliance. SHGs are linked to the Anandadhara scheme of the Government of West Bengal [40].

### Data collection

A semi-structured interview guide was developed and iteratively refined with input from faculty experts in climate change, public health, qualitative research, and the challenges in the Sundarbans (PN, GB, MS) (Appendix A). Questions were informed from a literature review on climate-related health impacts, sociodemographic and structural vulnerabilities, and disaster recovery strategies among cyclone-vulnerable regions globally, as well as the study team’s conceptual framework on cyclone-related health impacts in the Sundarbans. The draft guide was piloted with two stakeholders, and feedback was incorporated to improve alignment with the study’s research questions (Table 1).

Interviews focused on five a priori domains: (1) the ecological, geographic, and socioeconomic conditions predisposing the region to repeated cyclone impacts; (2) the immediate and long-term health, economic, infrastructural, and social impacts of cyclones; (3) existing response and recovery systems and capabilities; (4) structural barriers that hinder effective response and long-term recovery; and (5) community-led solutions for strengthening disaster response and recovery. Data collection was continued until thematic saturation was reached, defined as the point at which no new substantive concepts emerged in subsequent interviews. This determination was made in consultation with study team members experienced in qualitative research (PN, GB, MS). All interviews were audio-recorded via Zoom, transcribed via Sonix (Sonix, San Francisco, CA), verified for accuracy against audio recordings, and anonymized. Anonymized transcripts were uploaded to Dedoose (Dedoose, Manhattan Beach, CA) for coding and analysis. This study was approved by the Institutional Review Board of the University of California, Berkeley.

### Qualitative analysis

The analytic process proceeded via three stages: (1) initial deductive coding using initial a priori codes; (2) inductive development of new codes, themes, and subthemes that expanded and categorized the a priori domains; and (3) comparative analysis of thematic patterns and differences across stakeholders.

To ensure reliability and consistency, two coders (GB, PN) practiced applying an initial set of a priori codes and independently coded two transcripts in consultation with MS. This process enabled resolution of early discrepancies and calibration of an aligned, preliminary coding method. Thereafter, following frameworks by Kiger et al. [41], PN and GB manually reviewed and independently coded each transcript, meeting to discuss emerging concepts, inductively refine the codebook, and jointly formalize interrelated themes and subthemes [36]. Thereafter, a comparative analysis was conducted systematically. PN manually reviewed all excerpts and transferred each quotation into a master analytic spreadsheet per corresponding themes and subthemes. This chart served as the foundation for data synthesis and informed the organization of the thematic results after consensus was obtained with the study team. Reflexivity was prioritized throughout data collection, coding, and synthesis [42].

## Results

### Interview background

We conducted a total of 33 key informant interviews (KIIs) with diverse stakeholders, each lasting 15 to 65 minutes. All interviews were conducted with the aid of an English–Bengali translator and held at community spaces (e.g., schools, hospitals, Integrated Child Development Services (ICDS) centers, NGO offices, etc.). Interviews included (11) adult community members (drivers, farmers, shopkeeper, homemakers, members of Women’s SHG, and non-licensed village doctors); (7) frontline public health workers (ASHAs and AWWs); (3) elected local government officials (Gram Panchayat Pradhan); (4) appointed government officials; (2) mental health counselors; (1) professor of disaster management from a flood-prone adjacent state; and (5) senior leaders at a locally embedded NGO. Twenty participants (60.6%) were women—in part because all ground-level public health workers in the region are women. All participants had direct experience leading and/or coordinating cyclone response; and all participants except the NGO leaders, one mental health counselor, and the professor of disaster management were residents of cyclone-affected communities in Kultali and Gosaba.

Reflexive thematic analysis combined deductive and inductive approaches. We organized findings around five a priori domains, each aligned with the study’s core research questions: (1) the ecological, geographic, and socioeconomic conditions predisposing the region to repeated cyclone impacts; (2) the immediate and long-term health, economic, infrastructural, and social impacts of cyclones; (3) existing response and recovery systems and capabilities; (4) structural barriers that hinder effective response and long-term recovery; and (5) community-led solutions for strengthening disaster response and recovery.

## Section 1 | Contextual Vulnerabilities of the Sundarbans: Ecological, Geographic, and Socioeconomic Conditions

Participants identified ecological, geographic, and social conditions that exacerbated the Sundarbans’ vulnerability to impacts from repeated cyclones.

### 1.1 Ecological and geographic vulnerabilities in the sundarbans

**Figure 1:**
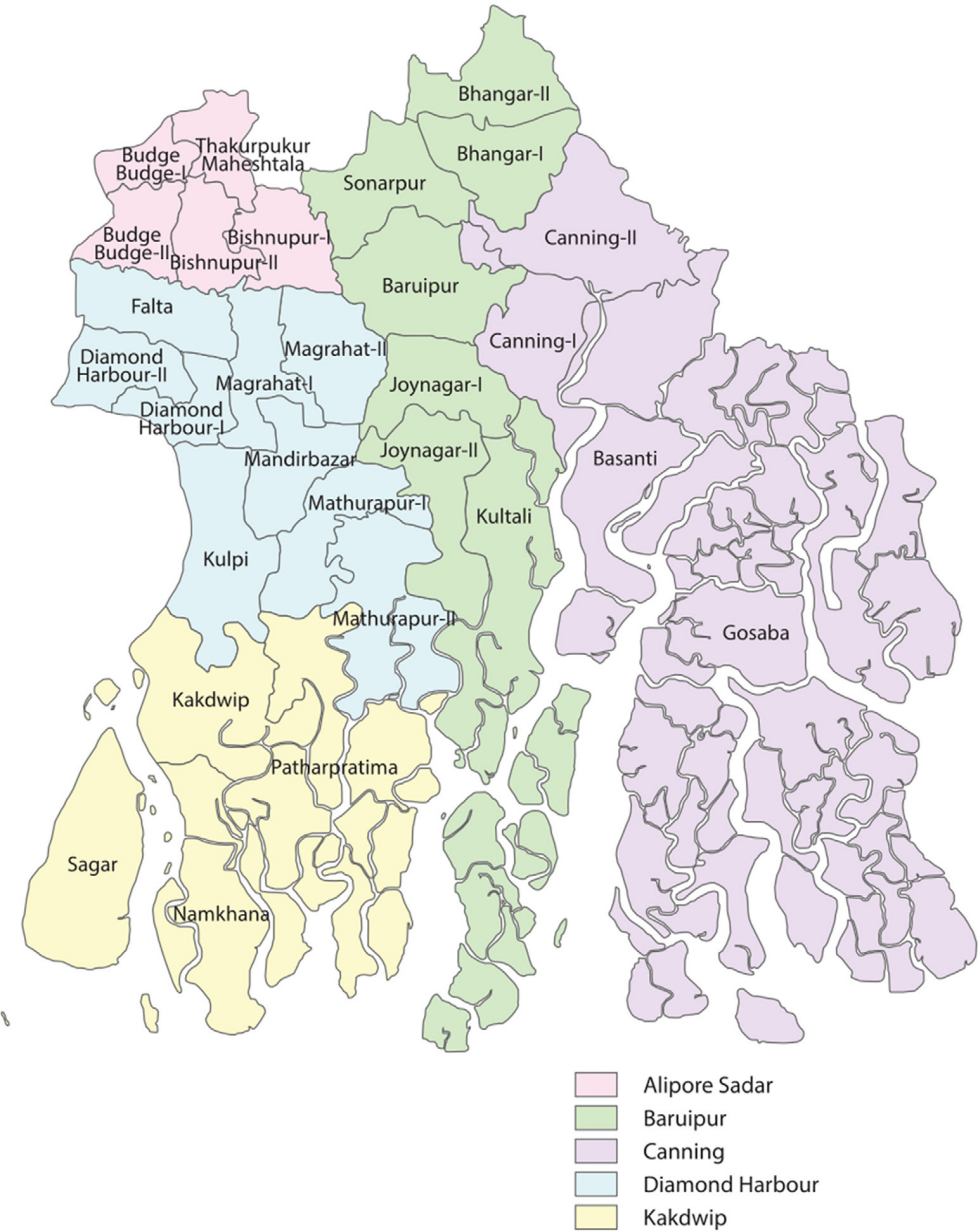
Map of South 24 Parganas showing Gosaba and Kultali, Sundarbans, West Bengal, India. *Source: Tanvir Anjum Adib / Wikimedia Commons, CC BY-SA 4.0*.

According to ASHAs and officials, the Sundarbans face rising sea levels, increasing temperatures, and more frequent, intense cyclones. Several community members described witnessing between four and seven flooding events annually, with at least six major storms causing significant harm in recent years. Gosaba and Kultali were identified by counselors as especially vulnerable blocks (Figure 1). Flanked by rivers on both sides, study participants reported that villages in these areas experienced disproportionately high rates of mental illness and suicide, driven by the repeated infrastructural damage and annual economic losses.

*“In the past few years, I’ve seen around six or seven major storms. Among them, cyclones like Amphan and Bulbul affected us the most—we had to move from one place to another, and many of our domestic animals died as the water levels rose. We suffered a lot.” (Member of Women’s Self-Help Group)*

*“There’s a big river behind Gopalganj (a neighborhood in Kultali), and during Amphan, the waves reached up to the first floor of houses—that’s why the destruction was so severe. Compared to our area, which is on higher land near the road, Gopalganj is much lower and more exposed. People here say, ‘If we’re struggling, imagine what Gopalganj went through.’ It’s surrounded by rivers on both sides, and one of them is very large. According to residents, the floodwaters rose to the first floor of homes.” (ASHA Worker)*

### 1.2 Socioeconomic precarity and healthcare workforce shortages

A significant portion of families in the Sundarbans rely on jobs in farming, fishing, driving, and honey collection, all climate-sensitive livelihoods disrupted for months to years after cyclonic events due to flooding, saline water intrusion, and infrastructural damage to roads and boats. Gram Panchayat officials highlight the lack of alternative livelihoods as a key barrier to recovery, leaving communities without income for months each year: *“Most of the people here are fishermen or farmers—they need alternative sources of income. There are no industries. If some industries or other income-generating programs were introduced, it would help a lot. Right now, there are no other options besides farming and fishing.” (Deputy Gram Panchayat Pradhan)*

While we could not quantitatively verify the figures, one official estimated, *“Out of 25,000 people in the block, about 17,500 are farmers. If you count families, that’s about 5,000 to 5,500 farming families. Only one percent is servicemen, and the rest are primarily fishermen or others in informal work.” (Deputy Gram Panchayat Pradhan)*

These workforce shortages extended into the health system. Participants repeatedly emphasized the scarcity of qualified healthcare providers across the Sundarbans. In Kultali, one mental health counselor reported being the sole provider for 36,000 children, adolescents, and adults and caring for 400 people per month. *“In this locality, there are adolescent-friendly health clinics, but no doctors are available—only nursing staff. To access a trained counselor or psychologist, there is only one for the entire block. I am posted here, and once a month a psychiatrist visits. Medicines are available, but not at the grassroots level. If anyone wants counseling, support, or medication, they have to come to the rural hospital.” (Mental Health Counselor for Children, Adolescents, and Adults)* This absence of health professionals was attributed to reluctance to work in remote, cyclone-prone regions.

## Section 2 | Impacts of Cyclones in the Sundarbans and Vulnerable Populations

**Figure 2:**
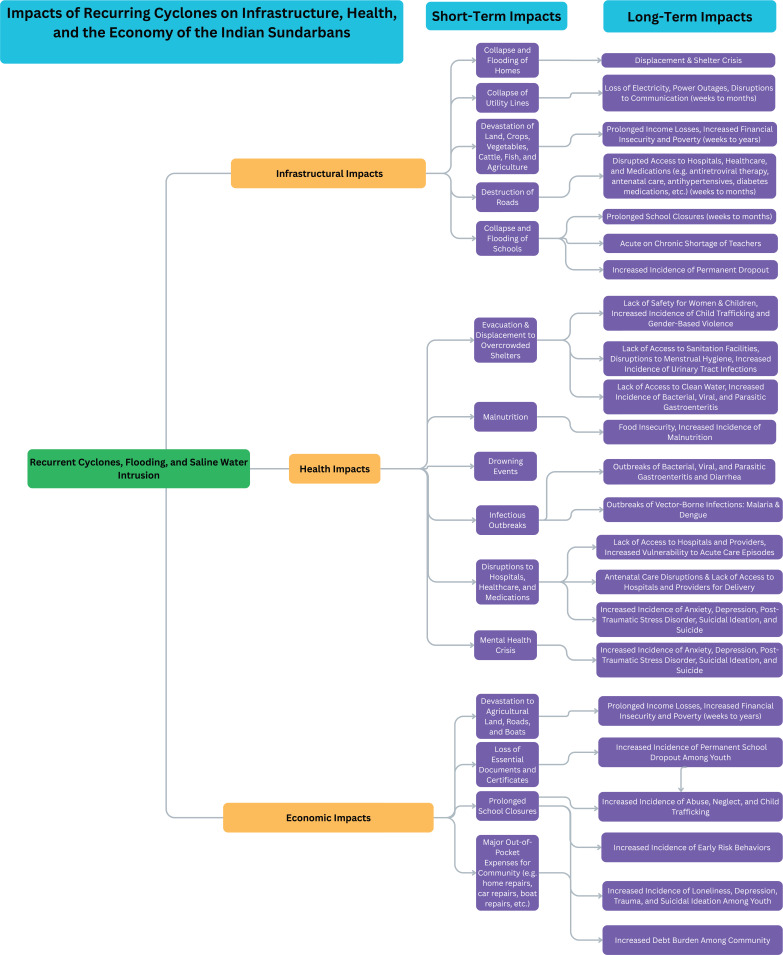
Conceptual model of cascading infrastructural, health, and economic impacts of recurrent cyclones in the Sundarbans.

Participants described immediate and long-term impacts of recurring cyclones in the Sundarbans through several interconnected lenses: displacement, infrastructural damage, economic insecurity, disruptions to children’s education, and compounding physical and mental health consequences (Figure 2).

### 2.1 Displacement and shelter crisis

During each cyclone, alerts issued by the Meteorological Department were relayed through Gram Panchayat officials and ASHAs. ASHAs then mobilized families to shelters via coordinated WhatsApp messaging and house-to-house visits, playing a key role in preparing families to gather legal documents, medicines, and dry food, and in supporting evacuation or shelter-in-place strategies for those unable or unwilling to leave.

For many residents, displacement has become a recurring and exhausting reality. As one shopkeeper from Gosaba reflected: *“I’ve seen this happen many times—our house gets swept away by rising river water. We have to take shelter in nearby relief centers, where we cook and survive with the supplies provided by the government. During those times, we live entirely in those shelters.” (Shopkeeper)*

A non-licensed village doctor shared a similar experience: *“Most of the time, our houses are swept away during floods. Whenever that happens, we have to leave and shift to another place. There have been several times when our homes were submerged by river water, and we had to rebuild new houses elsewhere.” (Non-Licensed Village Doctor)*

Gram Panchayat officials recalled the immense logistical challenges evacuating families to cyclone centers. *“The wind speed was 250 to 300 kilometers per hour. Saline water flooded the whole village…. Out of 7,500 families in this village, 5,500 were devastated…We tried to evacuate everyone to safe spaces and arrange food, shelter—everything. We coordinated with other departments who provided tarpaulin, food, and medicine.” (Deputy Gram Panchayat Pradhan)*

Despite these efforts, NGO leadership emphasized that evacuation and mass displacement were severely constrained by an insufficient number of shelters across the Sundarbans. Consequently, thousands of families were mobilized but forced to share overcrowded, unsanitary spaces that posed direct health risks, including heightened risk of diarrheal illness, poor menstrual hygiene management, and child trafficking.

### 2.2 Infrastructural damage

ASHA workers described how major cyclones such as Aila (2009) and Amphan (2020) caused extensive, large-scale infrastructural destruction, while smaller, recurring storms perpetuated infrastructural vulnerabilities.

Most homes were constructed from mud, asbestos, or tin and collapsed in the face of high winds and flooding. Rebuilding was described as a repetitive and futile cycle, with homes frequently destroyed and repaired, only to collapse in the next storm. One ASHA recounted her colleague’s reaction: *“She was really frightened because her house is a concrete house, but the roof is just metal. During Amphan, it was blown away. Suddenly there was no roof, and rain was coming in. She said, ‘We’ll all die.’” (ASHA Worker)*

Several ASHAs reiterated that paved roads broke down quickly under floodwaters, turning to thick mud and cutting off access to health services and relief efforts. NGO leaders reported that entire villages in Kultali lost electricity for up to three weeks after Amphan, leaving communities unable to power lights, charge phones, or communicate externally. *“Amphan had devastating effects across the entire area. For around two weeks—nearly 20 days—there was no electricity and no communication. Every place was in darkness.” (NGO Leader)*

### 2.3 Disruptions to livelihoods

NGO leaders reported that the flooding of agricultural lands, fishponds, and livestock was among the most destabilizing impacts. Salination could render soil infertile for months to years. Given that most households rely on cultivation for income, the loss of crops, fishponds, and cattle could trigger acute financial and food insecurity. As one elected official described: *“Almost all the men in our village are either fishermen or farmers. They’ve lost everything—their land has become infertile, and even the fish are gone. People are starving. They have no food, no water, no shelter. We’re searching for help from outside. If we get support during that time, it will make a huge difference.” (Gram Panchayat Pradhan)*

Another ASHA described watching her pond, once a key source of livelihood, deteriorate: *“Behind my house there are two big ponds with Hilsa fish—they’re very expensive. I could sell them in Calcutta for thousands of rupees. But there was no help to preserve them. They died. The Gram Panchayat couldn’t do anything.”* AWWs in Gosaba also emphasized how families, facing mounting out-of-pocket expenses, resorted to high-interest loans, some reportedly as high as ₹70,000.

### 2.4 Disruptions to education

AWWs, ASHAs, and mental health counselors consistently emphasized that recurring cyclones are a major cause of prolonged school closures that pose serious developmental, emotional, and safety risks for children. In 2024, flooding from recurring storms destroyed classrooms. Electrical systems failed, furniture was soaked, and windows were shattered. Beyond infrastructural damage, schools were repurposed as temporary shelters for displaced families or remained closed due to teacher shortages. As one ASHA explained: *“If ten teachers are needed, there may be only one. So how can they manage? Even after Class 10, there’s nothing in the school. For more than six months, children were going to school only to collect dry rations—rice, potatoes—not for education. Their attendance was marked, but there was no teaching. They just took the rations and came back home.” (ASHA Worker)* Without access to education for extended periods, school closures increased children’s risk of permanent dropout.

As one participant shared, *“During Cyclone Amphan, we distributed dry rations and nutritional supplements directly in the field, especially for children. But I noticed that their education was severely impacted by the disaster. Due to the lockdown and the cyclone, their schooling suffered even more. They were stuck at home most of the time and became increasingly dependent on smartphones.” (NGO Leader)*

One prominent barrier to continued education was the loss of essential documents. Flooding risked destroying educational certificates, preventing children from re-enrolling in school.

### 2.5 Health impacts and disruption to health services

The health consequences of these experiences were wide-ranging. These impacts precipitated outbreaks of infectious diseases, disrupted treatment for chronic conditions such as hypertension and diabetes, exacerbated malnutrition, and had lasting impacts on mental health.

One epidemiologist reported that overflowing latrines and contaminated water supplies led to spikes in diarrhea, urinary tract infections, malaria, dengue, drowning, and even death in the days following storms. Impassable roads disrupted access to antenatal care, contributing to anxiety among pregnant women who feared complications during delivery, according to a mental health counselor. People living with HIV (PLHIV) faced treatment disruptions and avoided shelters out of fear that public knowledge of their diagnosis could lead to discrimination or exclusion from cyclone shelters and essential resources. ASHAs reported rashes among infants, children, and parents from prolonged saltwater exposure, disrupted immunization schedules due to displacement, and gaps in malnutrition surveillance among families forced to migrate.

For many families, seeking care was simply not an option. Cyclones rendered existing health services inaccessible as fragile roads collapsed under flooding and a shortage of boats further isolated entire communities in Gosaba, Kultali, and surrounding blocks from hospitals for days to weeks. In the absence of formal care, families relied on non-licensed village doctors with limited capacity to manage complex or emergency needs. *“During Amphan, their house structures were so poor that they were totally devastated. They had no house at all. Can you imagine their situation? They were suffering from HIV and getting ART from the government hospital. But during Amphan, they couldn’t go to the hospital to access ART. And then what solution did we have for that? Mentally, they were totally depressed from the start.” (Physician and NGO Leader)*

One elected official explained, *“If something happens in the evening or at night, there’s nowhere nearby we can go for help. This is a mostly waterlogged area, and if someone is bitten by a snake or has a critical condition like a stroke, we have to travel to the nearest hospital—about 40 kilometers away—and even cross a river to get there.” (Deputy Gram Panchayat Pradhan)*

Another official with expertise in vector-borne disease control emphasized the compounding health risks: *“During storms, people suffer from severe diarrhea and mosquito-borne diseases like dengue and malaria. The roads become flooded, making it very difficult to take patients to the hospital, and boats are often unavailable because of high water levels. Pregnant women suffer the most, and nearby unlicensed doctors provide only initial support. Healthcare professionals and government doctors are unable to reach us.” (Appointed Government Official)*

Two non-licensed village doctors echoed these barriers.

*“The main problem we face is the poor condition of the roads. Most of the time, we have to travel by boat, but there are not enough boats in our area. To reach the hospital in Gosaba, we have to make a very long journey, which often makes it impossible for the sick or those needing treatment to get proper access.” (Non-Licensed Village Doctor)*

*“We face a lack of proper medicine, and those with serious health issues suffer the most. Many times we have to travel long distances to reach the hospital in an emergency. During that journey, people often die on the way, and families lose their loved ones.” (Non-Licensed Village Doctor)*

### 2.6 Malnutrition

Participants reported that malnutrition among children was a longstanding public health crisis in the Sundarbans, and cyclones further disrupted already fragile systems. AWWs, responsible for providing one reliable meal a day to children under age five, described how their place of work—ICDS centers—was frequently repurposed as emergency shelters following cyclones. As a result, children lost access to meals, and mothers lost access to nutrition counseling and lactation support for weeks to months.

As one AWW explained: *“Kids come here for nutrition…. The government made this scheme [ICDS] for that. So my job is to make sure they get the correct nutrition. I tried to save the food—I put it on the top shelf so the rice and food wouldn’t get ruined. But after the flooding, others had nowhere to go, so they came and stored their things there. My job was definitely hampered—I couldn’t give the children the nutrition they needed.” (AWW)*

Several AWWs observed increased desperation among children in shelters due to food insecurity: *“They’re already not eating properly. Many are malnourished. During Amphan and other cyclones, they end up in flood centers, struggling for food. The children start pushing, jumping over each other. That desperation turns into aggression. And still, they don’t have the nutrition or strength to cope. We’re trying our best to hold things together, but we don’t know how long we can.” (AWW)*

Disruptions in household income and school meal programs exacerbated pre-existing food insecurity.

### 2.7 Mental health

Frontline ASHAs and AWWs underscored that when these disruptions stretched over weeks and months, they were closely associated with long-term mental health consequences. One mental health counselor echoed this sentiment, describing a steady intensification of distress, with rising rates of trauma, depression, post-traumatic stress disorder (PTSD), and even suicide. She emphasized that children and wives exhibited signs of loneliness, depression, trauma, and even suicidal ideation, exacerbated by instability and trauma.

*“We often see mental health issues—depression, PTSD, and severe emotional stress—after every cyclone. Suicidal tendencies are very high here, and it’s clearly linked to climate change. It’s a serious issue. We’re working on it, and so is the government, but the impact is severe and deeply connected to climate-related events.” (Mental Health Counselor)*

*“There’s hardly any cultivation now because of water scarcity, so there’s no income. As a result, [men and] elders are migrating for work, while the younger generation and the wives are left behind. They have no one to talk to, and many are experiencing suicidal thoughts.” (Mental Health Counselor)*

AWWs also emphasized the emotional toll on children, noting that signs of trauma were visible and profound:

*“What I’ve noticed is that children go very quiet after these atrocities. We try to help them through drawing and toys, so that at least some happiness returns… [but] If you’re a kid and suddenly your parents are picking you up and running to a flood shelter in the middle of extreme rain and a cyclone, how would you feel? Terrified, right? That’s how it is for them. This fear runs so deep that it brings silence to their lives again and again. Sadness settles in, and happiness fades away.” (AWW)*

### 2.8 Vulnerable populations

Participants described how several populations—including farmers, women (including pregnant women), girls, PLHIV, and children—were especially vulnerable to these health, economic, and social impacts (Table 2). Farmers faced loss of land, livestock, and food security, leading to debt, migration, and mental distress. Women contended with reproductive health challenges, gender-based violence, and severe mental health harms. Pregnant women struggled to access antenatal care, placing themselves and their unborn children at risk. Children were vulnerable to abuse, school dropout, and increased phone and social media use. This was identified as a risk factor for trafficking, as predators would solicit children through social media platforms. PLHIV faced treatment disruptions and social exclusion.

**Table 2:** Stakeholder-identified vulnerable populations, risk factors, and impacts.

POPULATION	RISK FACTORS AND IMPACTS	EXEMPLARY QUOTES
Farmers	Economic Devastation: Farmers experienced extensive loss of fertile farmland, livestock, and income—leading to prolonged economic insecurity, significant loan burdens, and migration. Food Insecurity and Government Dependence: Farmers were dependent on prolonged government rations, leading to sustained food insecurity. Mental Health Strain: Prolonged economic hardship contributed to widespread emotional distress and increased risk of depression and suicide.	*“In our farming, we face a lot of challenges, such as flooding. All our fields are waterlogged.” (Farmer)* *“They lost their jobs, their cultivated lands—everything was destroyed. The level of destruction here is worse than in other areas. All the houses were gone after the dam broke. Many cattle, cows, dogs, and other animals drowned.” (ASHA Worker)* *“Most of the ponds became unusable, and the agricultural land remained uncultivable for 2 to 3 years after the saline water intrusion. The land became infertile. As a result, people lost their jobs, their livelihoods—everything. The Panchayat provided food rations so they could survive, and many became migrant laborers, leaving the area in search of work. The rations are still being provided to some families, but the quantity has decreased. Initially, the support was significant, but now it has been reduced.” (ASHA Worker)*
Women and Girls	Trafficking and Gender-Based Violence: Women faced disproportionately higher rates of trafficking and gender-based violence during and after displacement. Reproductive Health Challenges: After evacuation to cyclone centers, women were unable to maintain adequate menstrual hygiene due to limited space, lack of privacy, and insufficient access to sanitary products and clean water.	*“When people leave their homes and take shelter in common spaces, those places often become unsafe—especially for women and girls. Sometimes, trafficking even happens from within these shelters. Also, because the area is flooded, there are serious challenges related to hygiene, particularly menstrual hygiene for girls.”* *(NGO Leader)* *“Menstrual health and hygiene are severely affected. First, there’s the issue of supply—where will they get sanitary products? Even if they manage to get them, there’s the problem of disposal. Sometimes adolescents end up wearing the same pad for an entire day or longer, which increases the risk of infection. On top of that, shelter homes are often overcrowded and lack proper sanitation or washroom facilities, making it even harder to maintain hygienic practices.” (NGO Leader)* *“In cyclone shelters, there are often no separate spaces for men and women—everyone is lying together on the floor. In many cases, there are no separate toilets either. Because of menstrual taboos and the lack of privacy, women feel extremely uncomfortable and uncertain about how to manage their menstrual health and hygiene while surrounded by so many men.” (NGO Leader)* *“Child marriage and drowning are major issues here, along with mental health challenges. Human trafficking is also a serious problem. But above all, gender-based violence is something I deal with every single day.”* *(Mental Health Counselor)* *“Many girls couldn’t access sanitary napkins. Those using cloth often didn’t dry it properly, leading to a number of reproductive tract infections.” (NGO Leader)*
Pregnant Women	Antenatal Care Disruptions: Antenatal care was interrupted during cyclones due to flooded roads, damaged infrastructure, and overwhelmed health centers. Pregnant women were often unable to attend regular check-ups or receive necessary supplements and services. Delivery During Disasters: Pregnant women who went into labor during evacuation or while staying in shelters had decreased access to skilled attendants or proper delivery facilities. Anxiety and Worry: Pregnant women experienced heightened fear and uncertainty about their ability to reach health facilities during emergencies. Participants noted that many expressed deep concern for their unborn children and for surviving the pregnancy under such conditions.	*“It’s a terrifying experience—especially for women in their third trimester or nearing their delivery date. That’s when it’s crucial to monitor vitals like blood pressure, blood sugar, hemoglobin, and weight. It’s a very vulnerable time. We often get calls from the field saying, ‘We’re not getting these services—what should we do?’ This lack of access causes significant mental stress for pregnant women. And we know that stress during pregnancy can affect the entire delivery process. So yes, they become extremely anxious because of these gaps in care.” (NGO Leader)* *“For women whose delivery dates are approaching, we advise them to keep their phones charged so they can contact the ambulance or rescue services if needed. There’s a facility called the water ambulance, which is used for emergencies—especially for pregnant women who need to be transported for delivery. We’ve also spoken with the block authorities to ensure they can help mothers access this service when required.” (NGO Leader)*
People living with HIV (PLHIV)	Intergenerational Stigma and Social Exclusion: PLHIV, often men and migrant laborers, faced significant shame and stigma if their diagnosis became known within the community. Discrimination in Shared Spaces: Individuals with HIV were at-risk of exclusion from shared community resources such as flood shelters, evacuation centers, and places of worship. Treatment Disruption: Cyclones and displacement disrupted access to essential medications like ART. Food Insecurity: PLHIV experienced unmet nutritional needs during cyclones, compounding their health risks. Mental Health Distress: These challenges contributed to PLHIV experiencing panic, anxiety, and depression. Widowhood and Family Strain: In many families, the death of male PLHIV left women widowed, creating economic and emotional hardship.	*“They (PLHIV) had to travel to the hospital to get their antiretroviral therapy, but due to lack of transportation and limited financial resources, they were very panicked. They kept asking, ‘Will we get the medicine? Will we be able to travel to the hospital? What will we eat?’ They had seven days of rations—but what after that? That uncertainty haunted them the most. Some had built small kitchen gardens, but those were destroyed too. Everything was severely damaged. It deeply impacted their mental health, because these were things they had cared for over time—their home, no matter how small, their garden, their daily routine. And then, in a single day, everything was gone. That sense of panic came from the sudden disruption of what little stability they had.” (NGO Leader)* *“They isolate themselves from their peers. And if the villagers find out about a family’s HIV status, even today, they face discrimination. They’re often restricted from using shared resources like the village well or from worshipping in common community spaces.” (NGO Leader)* *“They were hesitant to go to community shelters because they feared discrimination. Sharing space with others who didn’t know about their HIV status felt risky—if people found out, they might not be allowed to stay. Since the shelter is a community resource, and these same communities often restrict PLHIV from using local ponds or worshipping at temples or mosques, there’s a real fear they would also be excluded from the shelter itself.” (NGO Leader)*
Children	Educational Disruption and Dropout: Prolonged school closures following cyclones left children without access to education for extended periods, increasing the risk of permanent dropout. Flooding also risked destroying essential educational certificates, preventing children from re-enrolling in school. Exposure to Abuse, Trafficking, and Neglect: With parents migrating to other cities for work, children were often left neglected. Unattended children—especially girls—faced higher risks of sexual exploitation, trafficking. Early Risky Behaviors: Children were also more likely to engage in early substance use, risky sexual activity, and excessive social media use—a risk factor for trafficking—as coping mechanisms or due to lack of oversight. Mental Health and Nutritional Risks: Children exhibited signs of loneliness, depression, trauma, and even suicidal ideation, exacerbated by instability and trauma. Disruptions in household income and school meal programs exacerbated pre-existing food insecurity.	*“What I’ve noticed is that many of the children have become extremely quiet and withdrawn after experiencing these disasters—what we call natural disasters…. They’re unable to express themselves. A kind of fearfulness is setting in. To help, my peers and I try to engage them through drawing and toys—anything that might bring a little happiness into their lives, to help break the silence.” (AWW)* *“After the disaster, [children] eventually dropped out. We’ve also seen other serious concerns—like adolescents, around 17 or 18 years old, turning to substance use.” (NGO Leader)* *“[Through social media, traffickers] say, ‘Come with us—we’ll give you a good job and pay you well.’ So people go. But when they arrive, they’re given some kind of medicine or drug, and then they’re trafficked to places like Delhi or Mumbai.” (ASHA Worker)* *“Officially, [parents] don’t say they’re selling their children. They say, ‘I’m marrying off my daughter’ or ‘You can adopt my child.’ But in reality, they ask for money—10,000 rupees, one lakh—and give the child away. Legally, exchanging money for adoption is illegal in India, but it still happens. So yes, they do sell their children, either through early marriage or so-called adoption.” (ASHA Worker)* *“Social media addiction is another major issue. Since no one is there to listen to them—parents are often absent or unavailable—children are given smartphones. They’re lonely and nobody is there to listen to them.” (Mental Health Counselor)*

## Section 3 | Existing Disaster Response Systems

Participants identified several core strengths that supported disaster response and recovery, ranging from early warning systems and cyclone center infrastructure to frontline efforts by ASHAs, local government, and NGOs. However, many of these resources were underinvested in and, as a result, were under profound strain.

### 3.1 Mangroves: natural ecological buffer

Participants emphasized that the Sundarbans’ growing ecological fragility aggravated these impacts. Coastal mangrove forests serve as critical ecological buffers. With upward-growing root systems that stabilize the soil, mangroves serve as natural barriers against storm surges and saline water intrusion. Yet participants reported that these ecosystems are rapidly degrading due to erosion and environmental pressures, leaving communities increasingly vulnerable to flooding. *“Since birth, I’ve witnessed many cyclones and natural calamities. Amphan was one of the worst. Our main protection comes from the trees—the mangroves. They protect us. The mangrove is like our mother. Without it, we would drown.” (Gram Panchayat Pradhan)*

They emphasized, *“If a wave like a tsunami comes, it will destroy everything—our concrete barriers, our big houses—everything. But what we can do is try to protect ourselves. We must start afforestation. We must save the trees. That may help nature protect us in return.” (Gram Panchayat Pradhan)*

There was a recognition of the importance of ecological protection across the community:

*“India is a land of huge population. So we are the reason for global warming, you know. And all these things. We are the cutting trees, destroying forests. Everything is happening and it is increasing day by day.” (NGO Leader)*

### 3.2 Asha workers and anganwadi workers: frontline responders and community health members

ASHA workers emerged as the critical link between communities and government systems throughout disaster response and recovery. ASHAs described being mobilized by Gram Panchayat officials in the wake of flooding to conduct real-time monitoring of disaster conditions. They described going out into the community to capture and send live updates, including photos of flooding and damaged infrastructure. They also described monitoring supplies, conducting needs assessments, and reporting gaps to authorities. *“Our duty is to take pictures and report how many people have been moved to the shelter, where they are, and what the situation is…. Every hour or two, we must report on the number of people relocated, the electricity status, whether the generator is working, and more. During cyclones, we are expected to handle everything, but we’re not given enough resources.” (ASHA Worker)*.

In Gosaba, ASHA workers opened their own homes to neighbors, providing food, safety, and guidance. *“One hundred and fifty people stayed in my house during the flood. My house is large, elevated, and doesn’t flood easily, so everyone comes here. I prepare potatoes, pumpkins, and other vegetables in advance because I know my neighbors will seek shelter with me. But I end up doing all the running around—cooking, helping—while others feel helpless. Even though this house has issues with the roof and feels unsafe, people still come here, because there’s nowhere else to go.” (ASHA Worker)*

Despite these burdens, ASHA workers embraced their role out of a sense of duty. *“It’s our job. We are very pro-India—patriotic by nature. My job is to help people, and I’m doing that. I’m an ASHA worker, so I do what I’m supposed to do. From newborns to the elderly, whoever needs support, I provide it. (ASHA Worker)*

Yet their testimonies reflected physical strain, emotional burnout, and lack of investment that may threaten the sustainability of this workforce. ASHA workers’ decision to open their homes during cyclones also highlighted the absence of institutional support: *“I brought people into my home out of a sense of humanity—but even it wasn’t easy. They used my toilet, my bathroom, and my bedroom. It became overwhelming. I couldn’t even keep my toilet clean, and I couldn’t express my frustration because I knew they were in need. I was torn—yes, I wanted to help, but there were limits.” (ASHA Worker)*

AWWs echoed overwhelming workloads, lack of recognition, and unpaid labor that extended beyond their official duties. *“As an ICDS worker, my job is to provide proper nutrition to children, educate them, and counsel adolescent girls. But in reality, I end up doing far more than what my role requires.” (AWW)* AWW workers also described using their own funding to support others. *“Sometimes I don’t even receive my full salary, because part of it goes toward feeding and educating the children who come to me for support.” (AWW)*

### 3.3 Early warning and cyclone centers infrastructure

Participants praised the reliability of the Sundarbans’ early warning systems as a central strength of the region’s response system, which has improved significantly since cyclone Aila. Cyclone centers were also broadly recognized as vital lifelines, offering shelter, food, water, medicine, and other essentials during cyclones. These multi-story structures are financially self-sustaining, serving as venues for weddings, ceremonies, and community gatherings during non-disaster periods to generate revenue for maintenance and disaster preparedness. One ASHA explained: *“The cyclone center is a two- or three-story building. On the ground floor, people keep their cattle—cows, goats, even roosters. The upper floors have separate spaces for women, men, and children. They provide everything needed—medicines, sanitary napkins, whatever is necessary. They take care of all of it.” (ASHA Worker)*

However, existing centers were overcrowded, lacked privacy, and posed safety risks for women and girls. Participants echoed incidences of gender-based violence and challenges managing their reproductive health. In Kultali, there are 7 cyclone centers for 229,053 people; in Gosaba, there are 10 cyclone centers for 246,598 people, according to NGO leadership with access to government reporting [43, 44]. One ASHA reported: *“One center is supposed to support 5,000 people, but in reality, it’s serving around 12,000. We’re seeing many cases of depression. People are deeply distressed—they say they’re depressed because they can’t care for their families or their children. They cry and ask, ‘What will happen to us?’ They are saying we are in depression.” (ASHA Worker)*

### 3.4 Gram panchayat-level services

Gram Panchayat officials described prioritizing post-disaster distribution of food, medicine, water, and essential supplies and highlighted the state’s deployment of mobile water trucks capable of producing up to 35 pouches of clean water per minute. These were distributed through local networks. To mitigate sanitation risks, local authorities installed temporary pit latrines and coordinated the delivery of antidiarrheal and antibiotic medications to cyclone centers. Officials acknowledged that such latrines were not ideal but prevented open defecation and contamination of public water sources, such as ponds. Despite these efforts, community members reported that services were often delayed or insufficient.

### 3.5 NGO-based services

NGO leaders view their role as strategic safety nets addressing service gaps for vulnerable populations. They described support they offered during a cyclone, *“We provided awareness about the present situation and weather to help reduce their panic. They were panicking, so we provided accurate information and knowledge to help them stay calm and take logical action…. Thereafter, provide information about where they can access government services—such as housing, shelter, treatment—and where free camps and health checkups are being conducted.” (NGO Leader)*

NGOs also supported training of ASHAs and AWWs on birth preparedness during disasters and advised pregnant women to keep phones charged, maintain contact with block authorities, and utilize emergency transport services if delivery becomes imminent during flooding.

To ensure continuity of ART during storms, one participant shared: *“We support people living with HIV by making sure they can access medications and nutritional support…. We used to work with people living with HIV, and during Cyclone Amphan we distributed dry rations and nutritional supplements directly in the field…. We also advocated to the government so ART could be made available at local health centers. Otherwise, they would have to travel to the district hospital, which becomes impossible during flooding.” (Physician and NGO Leader)*

NGO leadership also attempted offering phone-based counseling on menstrual hygiene and infection prevention to adolescents during disasters. *“Since our workers couldn’t always reach them because of waterlogging, we educated them by phone on how to manage their hygiene during the disaster.” (NGO Leader)*

Lastly, NGO leadership emphasized subsidizing funding for home repairs and proactively checking on community safety. *“Our fieldworkers visit people in their homes and follow up with phone calls. During COVID and Amphan, when phone lines didn’t work and we couldn’t reach many people, we went directly into the field to check whether they were in a safe space.” (NGO Leader)*

## Section 4 | Structural Barriers: Neglect and Chronic Underinvestment

Participants voiced deep frustration with chronic underinvestment in infrastructure and essential services that delayed cyclone recovery and perpetuated vulnerabilities and health impacts.

Participants highlighted the need for robust social services and policies to address persistent infrastructural vulnerabilities. Schools had asbestos roofs, poor ventilation, and visible disrepair. Homes were primarily constructed from asbestos, mud, and “flimsy wood,” materials prone to leakages and collapse; homes with concrete roofs were also flood-prone due to their low ground clearance. Such infrastructure was very vulnerable to the impacts of recurrent cyclones. Villages received only 90–110 volts, far below the standard 220 volts required to power fans, lamps, and devices. This led to frequent outages. During interviews in Gosaba for this research, the power failed three times in just two hours at a local site. Roads in both Gosaba and Kultali were narrow, scarce, and frequently damaged by floodwaters. The number of cyclone shelters and health infrastructure was also inadequate.

One stakeholder referenced challenges associated with the ICDS nutrition program in the region as an example of broader, persistent underinvestment in social services. An NGO leader reported that, of 458 ICDS centers in Gosaba, only 160 (35%) were functioning in concrete buildings; 30 to 40 were vacant, and most operated outdoors under trees, exposing mothers, infants, and AWWs to extreme heat and rain. Services were suspended for weeks to months after cyclones, contributing to malnutrition. “*There’s heavy rain in August. …children are unable or unwilling to come to us. We can’t even ask the children to come then, because we have to conduct everything under a tree. So how are we supposed to provide nutrition on a cyclonic day—how can we feed them like that?” (AWW)*

Another stakeholder identified a suite of implementation barriers that undermine planning and policy execution. *“Another major problem is data accuracy. For example, they might report that electricity is available when it’s actually not.”*

One stakeholder noted that existing response and recovery systems were reactive and short-term, leaving communities vulnerable to repeated harm. *“There’s no sustainable or long-term plan in place. There’s no systematic approach to prevent or withstand future calamities. The focus is mainly on immediate response—things like generating awareness, helping people protect their documents, and offering short-term support during and after the disaster. But these are temporary interventions, not lasting solutions.”*

NGO leaders and community members called for stronger government action to mitigate the impacts of cyclones. In contrast, several local officials emphasized the supremacy of nature and the need for self-reliance and community-wide training over state intervention. One official argued: *“We are tiny in front of nature. Nature is the ultimate force. Concrete can’t save us—big buildings, large barrages made of concrete, they cannot protect us. Only if we support nature will nature protect us. We need training on how to protect ourselves during natural calamities. Everyone in our village should be equipped with the knowledge and capacity to act during cyclones or other disasters. If there is advanced technology that can help us prepare and respond, we should have access to that too. But again, I want to emphasize: big buildings and concrete won’t save us.”*

## Section 5 | Community-Generated Recommendations for Policymakers

During interviews, we sought to understand what community members and stakeholders wanted policymakers to prioritize. As participants shared their recommendations—rooted in lived experience—addressing both immediate needs and long-term strategies to strengthen disaster preparedness, health access, and climate resilience, these ideas were subsequently discussed and refined with additional community stakeholders.

### 5.1 Immediate preparedness measures

One respondent recommended urgent, low-cost interventions that could be implemented immediately to strengthen disaster preparedness. Communities should be pre-equipped with chlorine tablets, clean water, and dry food, as government relief often arrives only after several days. Schools could establish systems to back up or digitize students’ essential documents to prevent permanent loss during disasters. For farmers, secure seed storage is critical, since cultivation is not possible for months following a cyclone. Additionally, at least one mental health counselor should be stationed at each PHC, where none are currently posted.

### 5.2 Community capacity building: training and education

NGO leaders and government officials emphasized that lasting resilience requires infrastructure, policy, and financing. Yet their responses also highlighted a critical need to equip communities with knowledge, habits, and skills to adapt to a changing climate. They outlined the importance of training community members to proactively safeguard essential documents before cyclones; fostering environmental awareness from an early age, much like basic hygiene or safety habits. ASHAs also identified drowning as a major risk during floods and called for survival training and swimming instruction. Dedicated environmental education at a young age was highlighted as a way to engage the community.

*“We should protect our environment—and that value should be taught to children from a very young age. Only then can we truly protect it. Let me give a simple example: toilet training. It’s something that starts in early childhood. In the same way, environmental awareness and care should become a basic practice from childhood. If we take small steps to protect our surroundings, those actions will give back to us in many ways. If we want our own well-being—physically, mentally, and socially—we must be more careful with the environment. And the habit of caring for it should start early. We can’t easily develop new habits in adulthood, but we can incorporate them in childhood with much more ease. That’s why environmental education and practice must begin at a young age.” (Mental Health Counselor)*

### 5.3 Policy recommendations to invest in livelihoods and simultaneously promote cyclone resilience

Across interviews, the need for a more cyclone-resilient economy emerged as one of the community’s most pressing priorities. Given that most families currently rely on cyclone-sensitive livelihoods, stakeholders emphasized that protecting existing livelihoods must be a core component of disaster response policy. Participants called for insurance coverage, compensation schemes, and infrastructural safeguards to help communities—especially farmers, drivers, and fishermen—recover from recurrent economic losses. *“When fishermen go out to sea and their launches, ferries, or trawlers are damaged by cyclones, they should be covered by insurance. But beyond insurance, livelihoods must be fundamentally protected through policy. It’s not just about compensating loss—it’s about ensuring sustainable, long-term security. These protections must be established and maintained by the government.” (Professor of Disaster Management)*

At the same time, Gram Panchayat officials, NGO leaders, ASHAs, and AWWs recognized the region’s overreliance on cyclone-vulnerable occupations and called for investments in livelihood diversification. Participants proposed expanding employment programs, industry activity, and vocational training to provide families with stable, sustainable livelihoods. *“People here are always in search of livelihoods. Once their basic livelihood needs are met, then they can devote their minds and energy to other aspects of life. Here, our people are still struggling for the minimum: toilets, roads, school buildings. That’s why our priorities are different.” (Professor of Disaster Management)*

In discussions with Gram Panchayat officials, who spoke passionately about the protective value of mangroves, nature-based livelihood strategies emerged as potential solutions to the region’s overlapping economic and ecological challenges. One recommendation was the development of mangrove reforestation programs that would provide temporary or long-term employment while simultaneously fortifying the Sundarbans’ most vital natural defense against flooding and salinization. Two officials emphasized:

*“Mangrove reforestation would be great—not just because of the planting itself, but because it makes nature more vibrant and full of oxygen. It makes the environment more beautiful. It also helps prevent soil erosion and protects us from cyclones. And if it also creates employment, then it would be truly beneficial for everyone.” (Executive Assistant to Gram Panchayat Pradhan)*

*“These villages need stronger embankments to prevent water from entering. More trees, more mangroves.” (Gram Panchayat Pradhan)*

*“To save our future generations, it’s [necessary] to plant more trees…. Government should take more initiative to plant trees and renovate these rivers…. That is the only way we can have a good future for our environment.” (Deputy Gram Panchayat Pradhan)*

To scale such initiatives, a disaster management expert recommended a series of policy reforms to mobilize private sector investment in the Sundarbans. Chief among these was amending India’s Corporate Social Responsibility (CSR) law, which currently limits companies to spending CSR within a 10-kilometer radius of their operations. *“Under current rules, private companies must spend 2% of their annual profits on social development. However, there’s a loophole in the policy. CSR funds must be spent within a 10-kilometer radius of where the company operates. For example, if a company is located in Kolkata, it won’t typically sponsor a program in the Sundarbans, which is 50–60 kilometers away. That’s because it falls outside the official 10-kilometer zone. This restriction prevents companies from supporting critical work in more remote, underserved areas. That loophole in the CSR policy needs to be removed.” (Professor of Disaster Management)*

The expert also called for the creation of targeted climate financing policies with explicit tax incentives to encourage private sector investment in environmental and disaster resilience projects, such as mangrove reforestation and climate resilient infrastructure. *“There’s also no targeted policy or tax incentive specifically aimed at environmental or climate-related initiatives. That needs to change.” (Professor of Disaster Management)*

From this perspective, effective policy reforms could catalyze private sector contributions to climate resilience, enabling initiatives that are both environmentally sustainable and economically empowering for vulnerable communities in the Sundarbans.

When these ideas were shared and tested with other community stakeholders—including ASHAs, AWWs, and NGO stakeholders—there was broad agreement that private sector investment and policy reforms were essential to developing and scaling climate-resilient livelihood programs. However, participants equally stressed that sustainable development cannot depend solely on external actors and that local governance bodies had to be empowered to lead effectively.

### 5.4 Long-term infrastructural and health investments

Participants identified several investment priorities to reduce vulnerability, sustain health and education access, and foster long-term recovery and development. These included constructing hospitals in the Sundarbans to address the absence of nearby medical care; renovating old ponds to restore their fertility for fishing and irrigation, thereby supporting livelihoods; strengthening and expanding roadways, which would prevent disruptions to health services, schools, and markets during floods; replacing mud-based homes with concrete structures to prevent the collapse of homes, displacement, and damage during storms; building concrete-based barrages along coastlines to prevent saltwater intrusion and protect village perimeters; and establishing mental health centers to provide trauma care after disasters.

As one driver reflected, *“I fear that one day, during a cyclone, our village might no longer be livable. My hope is that we’ll have proper medical facilities, safe shelters for flood-affected people, clean water, enough medicine, and protection for our children’s future.” (Driver)*

Farmers and a disaster management expert echoed this call for investment in secure shelter and healthcare access:

*“If we get a secure place where we can stay—shelter or proper healthcare center where we can have and get proper access to health services—that would be more beneficial and needed in that situation.” (Farmer)*

*“If we have proper, wider roads, it would be easier for us to travel to the nearest hospital. If we have bigger hospitals or health centers in our local area, more people could get access to proper healthcare services.” (Farmer)*

*“The government should establish strong institutions in our country to provide mental health support to victims of cyclones and other disasters. There needs to be a clear plan to create dedicated centers where survivors can receive moral support, counseling, and training—along with awareness on mental trauma, emotional wellbeing, and other psychological needs.” (Professor of Disaster Management)*

NGO stakeholders emphasized that Gram Panchayat officials should be equipped to drive climate resilience by integrating localized Sustainable Development Goal (SDG) indicators into the Gram Panchayat Development Plans to ensure disaster preparedness and long-term recovery are embedded into local planning and budget allocation.

*“The Government of India has aligned development planning with the Sustainable Development Goals (SDGs). So when Panchayats develop their plans, they should include localized SDG indicators—especially those relevant to disaster response and preparedness. That way, they know which indicators to focus on, what actions to take, and why to invest resources accordingly.” This approach not only aligns with national priorities but ensures local ownership and accountability in long-term resilience planning. (NGO Leader)*

## Discussion

This study investigates the significant and cascading impacts of recurrent cyclones on the physical and mental health, educational opportunities, livelihoods, infrastructure, and overall well-being of coastal communities in the Sundarbans—one of the world’s most climate-vulnerable regions. It also highlights barriers to recovery and long-term development. We deliberately employed a deductive and inductive qualitative methodology centered on the voices of community-embedded stakeholders, including community members, frontline public health workers, local government officials (Gram Panchayat Pradhan), NGO stakeholders, and mental health counselors, to illuminate the lived realities, perspectives, and recommendations of those directly affected and responsible for disaster response.

Participants identified key resources within the existing disaster response system, including reliable early warning community mobilization protocols; multi-purpose cyclone centers that provided shelter, food, water, medicines, and temporary safety; tireless frontline public health workers like ASHAs and AWWs, who supported families in navigating impacts and opened their personal homes when formal shelters were overcrowded or inaccessible; and targeted NGO support for vulnerable populations (Table 2), which complemented government response and filled critical service gaps. Coastal mangrove forests were a source of tremendous pride and sense of belonging for the community, and served as foundational natural barriers against saline water intrusion during cyclones.

Yet, despite these resources, the overarching narrative was one of cyclical entrapment. Communities remain caught in a recurring cycle of destruction and inadequate recovery, with each cyclone causing repeated and compounding infrastructural damage, livelihood insecurity, prolonged educational disruptions, and physical and mental health harms. This persistent instability visibly forced families to focus their lives around immediate survival—securing basic necessities like food, water, shelter, healthcare, education, and safety to fulfill the first two foundational tiers of Maslow’s hierarchy of needs [45]. These needs remain unfulfilled due to a lack of appropriate policy and robust social services. Until these basic needs are met, aspirations for safety, education, empowerment, social advancement, self-actualization, and long-term development will remain unattainable.

In recent years, the Sundarbans have faced more frequent and severe cyclones, each triggering ecological, economic, infrastructural, and health crises. Flooding and saline water intrusion, exacerbated by mangrove degradation, devastate agricultural land, livestock, fishponds, crops, and boats, disrupting farming and fishing, the region’s economic backbone. Homes of mud, asbestos, or hay collapse; roads turn to impassable muds, preventing access to hospitals, schools, and markets for weeks. Since Cyclone Aila (2009), evacuation protocols have improved, but shelter shortages still force thousands into overcrowded, unsanitary spaces, heightening the risk of gender-based violence, urinary tract infections, and trafficking. Outbreaks of diarrhea, dengue, malaria, and tuberculosis follow, while pregnant women, PLHIV, and people with chronic conditions lose access to essential medications. Recovery was characterized by prolonged hardship. Families return to looted homes, lost school certificates, and mounting debt from high-interest loans. Farmers rely on food rations; many parents migrate for work, leaving children with elderly relatives. Prolonged school and ICDS closures disrupt education and meals, with reopened schools often serving only as ration sites. Closures exacerbate malnutrition, dropout, abuse, trafficking, early marriage, sexual exploitation, and early substance-use—risks compounded by excessive unstructured time, social media use, and predatory contact by traffickers. Mental health counselors report sharp rising distress, trauma, depression, PTSD, and suicide, especially in river-flanked areas, echoing prior findings on deliberate self-harm in the Sundarbans [46]. These impacts fall heaviest on ASHAs, farmers, children, women (including pregnant women), and PLHIV.

Our findings suggest that at the core of these recurring health crises is a web of interconnected ecological, structural, and sociodemographic vulnerabilities. Ecologically, the Sundarbans face accelerating mangrove degradation, rising temperatures, and more frequent, intense storms [20, 47]. Structurally, local officials were described as offering only partial solutions—such as poorly constructed roads or repeated reconstruction of homes using mud and asbestos instead of more resilient materials. Economically, most households rely on climate-sensitive livelihoods, such as agriculture, fishing, and honey collection, with few viable alternatives. Fragile educational systems further constrain generational mobility and opportunity. Chronic shortages of doctors, mental health counselors, and teachers limit access to essential medical services and undermine recovery. These vulnerabilities are compounded by diverging perspectives on responsibility: several government officials framed nature as supreme and advocated for individual preparedness over systemic investment, effectively shifting the burden of resilience onto the families most affected by cyclones. Together, these dynamics constitute what Paul Farmer described as structural violence: political and social arrangements that systematically constrain basic human needs, invisibly perpetuating vulnerability, and obstructing community development and well-being without accountability [48].

Community stakeholders outlined priorities for strengthening cyclone preparedness and recovery. Immediate, low-cost measures include pre-positioning chlorine tablets, clean water, and dry food; digitizing and safeguarding students’ essential certificates; establishing secure seed storage for farmers; and ensuring at least one mental health counselor at every PHC. Longer-term strategies focused on protecting and diversifying livelihoods, reforming policy and financing, and investing in resilient infrastructure, especially hospitals, roads, and boats to expand and sustain access to care. The absence of nearby hospitals and reliable roads has transformed past medical conditions, acute illness, childbirth, and injuries into life-threatening emergencies during disasters; establishing accessible, cyclone-resilient medical facilities and expanding the workforce of available health professionals emerged as one of the community’s strongest and most consistent demands. Recommendations also included providing insurance coverage and compensation schemes for cyclone-sensitive occupations, and expanding employment programs, vocational training, and local industries. Mangrove reforestation was highlighted as a source of community pride, a livelihood opportunity, and a critical ecological defense. Participants called for amending India’s CSR law to enable private sector investment beyond a 10-kilometer radius, introducing tax incentives for climate-resilient initiatives, and empowering Gram Panchayats to lead local resilience planning. Such policy reforms would support investments such as building hospitals, expanding and climate-proofing roads, renovating ponds, constructing resilient housing, and establishing dedicated mental health centers.

To date, the health, social, economic, and infrastructural consequences of cyclones in the Sundarbans remain largely underexamined. This investigation advances the literature in several ways. It is the first to apply qualitative, interview-based inquiry to examine impacts, vulnerabilities, resources, and policy and investment gaps; to draw from a diverse cohort of stakeholders with direct experience living through and responding to cyclones; and to generate stakeholder-informed recommendations. This multi-level perspective provides a deeper, more actionable understanding of the unique hardships facing the Sundarbans and opportunities to foster climate resilience and long-term recovery.

This study has several limitations. First, it is subject to selection bias, as interviewees were identified through a partner NGO and may have had a particular interest in cyclone response and recovery. This potentially skewed results toward more critical perspectives or recommendation-oriented viewpoints. Second, all participants were recruited from Kultali and Gosaba. While these are among the most vulnerable areas of the region, the findings may not be generalizable to the entire Sundarbans and may reflect regional or geographic biases, including heightened awareness of specific infrastructural and policy limitations. We sought to mitigate these limitations by recruiting a wide range of participants, interviewing until thematic saturation was reached, eliciting reflections on a broad spectrum of experiences, beliefs, and observations, in accordance with COREQ guidelines [49].

If implemented, these targeted interventions could mitigate the health, educational, economic, ecological, and infrastructural impacts of cyclones, laying the groundwork for sustained recovery and equitable development in the Sundarbans. The Sundarbans’ cyclone-related challenges are a microcosm of the threats climate change poses to vulnerable, impoverished communities around the world. Prolonged hardship characterized by delayed investment, neglect, administrative failures, conditions for inadequate policy response, and sustained suffering offers a sobering preview of what coastal communities across the Global South may continue to face as disasters grow more frequent and severe, and as COP negotiations continue to fall short on mitigation, adaptation, and loss and damage financing [50–52].

As governments and world leaders convene at international climate summits and navigate the emerging impacts of the climate crisis, the experiences of coastline communities like the Sundarbans must be positioned at the center of climate change and health discourse. The Sundarbans lies at the frontlines of the climate crisis and exemplifies how the health impacts of climate change disproportionately harm those who have contributed least to its cause, while threatening to erase decades of progress in global health, sustainable development, and poverty reduction. As those in the Sundarbans continue to bear the unjust cost of high-emission economies, their perspectives demand recognition within global climate policy and beckon collective action from the global community.

## Conclusion

This investigation explores how recurrent cyclones undermine physical and mental health, livelihoods, infrastructure, and long-term development in the Sundarbans, and generates stakeholder-informed recommendations for policymakers.

Participants consistently emphasized the fragility of essential systems—degrading mangroves; homes, roads, and electricity lines prone to collapse; shortages of medications, health facilities, health professionals, and cyclone centers; and overreliance on climate-sensitive livelihoods resulting in profound income losses—exacerbated by chronic underinvestment and neglect. While frontline responders such as ASHA workers, Anganwadi workers, NGOs, and community members play indispensable roles in navigating cyclone response and recovery, they do so under conditions of precarity and strain, and communities remain entrapped in cyclical destruction and inadequate recovery.

Proposed recommendations ranged from immediate, low-cost measures (e.g., digitized educational certificates, clean water, dry food, secure seed storage) to longer-term strategies such as livelihood protection and diversification, expanded insurance and employment schemes, investments in hospitals, roads, and resilient infrastructure, mangrove reforestation programs, and policy reforms—including CSR law amendments and tax incentives—to mobilize investments in the region.

The suffering of those in the Sundarbans exemplifies the unjust health impacts of the climate crisis. Collective commitment to address coastline communities’ needs is a measure of our collective resolve to confront climate injustice. Solutions centering on the well-being and vitality of the people and the ecology of the Sundarbans offer an opportunity to secure health, dignity, and the realization of their full potential.

## Data Availability

The qualitative data generated during this study are not publicly available in accordance with the study’s Institutional Review Board (IRB) protocol. De-identified excerpts supporting the findings are included in the article.

## Additional Files

The additional files for this article can be found as follows:

Supplementary Material. Figure 1.

Supplementary Appendix A. Semi-Structured Interview Guide.

## References

[r1] CDC. Vector-borne diseases. Climate and Health. Published 2025. Accessed June 17, 2025. https://www.cdc.gov/climate-health/php/effects/vectors.html.

[r2] CDC. Temperature extremes. Climate and Health. Published 2025. Accessed June 17, 2025. https://www.cdc.gov/climate-health/php/effects/temperature-extremes.html.

[r3] CDC. Precipitation extremes. Climate and Health. Published 2025. Accessed June 17, 2025. https://www.cdc.gov/climate-health/php/effects/precipitation-extremes.html.

[r4] CDC. Mental health and stress-related disorders. Climate and Health. Published 2025. Accessed June 17, 2025. https://www.cdc.gov/climate-health/php/effects/mental-health-disorders.html.

[r5] CDC. Air pollution. Climate and Health. Published 2025. Accessed June 17, 2025. https://www.cdc.gov/climate-health/php/effects/air-pollution.html.

[r6] CDC. Food and waterborne diarrheal disease. Climate and Health. Published 2025. Accessed June 17, 2025. https://www.cdc.gov/climate-health/php/effects/food_waterborne.html.

[r7] CDC. Food security. Climate and Health. Published 2025. Accessed June 17, 2025. https://www.cdc.gov/climate-health/php/effects/food-security.html.

[r8] CDC. Allergens and pollen. Climate and Health. Published 2025. Accessed June 17, 2025. https://www.cdc.gov/climate-health/php/effects/allergens-and-pollen.html.

[r9] Sun L. CDC warns of increased dengue fever risk in US. Washington Post. Published June 25, 2024. Accessed July 13, 2024. https://www.washingtonpost.com/health/2024/06/25/dengue-fever-outbreak-warning-mosquito/.

[r10] Romanello M, Walawender M, Hsu S-C, et al. The 2024 report of the Lancet Countdown on health and climate change: Facing record-breaking threats from delayed action. Lancet. 2024;404(10465):1847–1896. doi:10.1016/S0140-6736(24)01822-1. 39488222 PMC7616816

[r11] Gore T. Confronting Carbon Inequality: Putting Climate Justice at the Heart of the COVID-19 Recovery. Oxfam; 2020. Accessed July 17, 2025. https://oxfamilibrary.openrepository.com/bitstream/handle/10546/621052/mb-confronting-carbon-inequality-210920-en.pdf.

[r12] Eckstein D, Künzel V, Schäfer L. Global Climate Risk Index 2021: Who Suffers Most from Extreme Weather Events? Weather-Related Loss Events in 2019 and 2000–2019. Germanwatch; 2021. Accessed January 26, 2025. https://www.developmentaid.org/api/frontend/cms/file/2021/03/Global-Climate-Risk-Index-2021_1.pdf.

[r13] XDI Cross Dependency Initiative. Gross domestic climate risk ranking of 2,600+ territories. Published 2024. Accessed July 17, 2025. https://archive.xdi.systems/gross-domestic-risk-dataset/.

[r14] Wan W, Feng X, Liu Q, et al. Effect of landfall location and coastal topography on surge response in the Northern Bay of Bengal. Reg Stud Mar Sci. 2020;40:101476. doi:10.1016/j.rsma.2020.101476.

[r15] Tropical cyclones in the Indian ocean. Climate Research Lab @ IITM. Published 2022. Accessed August 10, 2025. https://www.climate.rocksea.org/research/tropical-cyclones-indian-ocean/.

[r16] Dube SK, Poulose J, Rao AD. Numerical simulation of storm surge associated with severe cyclonic storms in the Bay of Bengal during 2008-11. Mausam. 2013;64(1):193–202.

[r17] Kossin JP, Knapp KR, Olander TL, et al. Global increase in major tropical cyclone exceedance probability over the past four decades. Proc Natl Acad Sci U S A. 2020;117(22):11975–11980. doi:10.1073/pnas.1920849117. 32424081 PMC7275711

[r18] Acharya A. Restoring mangroves in non-protected areas of the Sundarbans. The Nature Conservancy. Published 2025. Accessed August 11, 2025. https://www.nature.org/en-us/about-us/where-we-work/india/our-priorities/restore-mangroves-sundarbans/.

[r19] Jabir A-A, Hasan GMJ, Anam MM. Correlation between temperature, sea level rise and land loss: An assessment along the Sundarbans coast. J King Saud Univ - Eng Sci. 2021;35:141–150. doi:10.1016/j.jksues.2021.07.012.

[r20] Sahana M, Rehman S, Paul AK, et al. Assessing socio-economic vulnerability to climate change-induced disasters: Evidence from Sundarban Biosphere Reserve, India. Geol Ecol Landscapes. 2021;5(1):40–52. https://www.tandfonline.com/doi/full/10.1080/24749508.2019.1700670.

[r21] UNESCO World Heritage Centre. Saving the lives of tigers and people in the Sundarbans. Published 2020. Accessed August 10, 2025. https://whc.unesco.org/en/news/2154.

[r22] Mishra M, Acharyya T, Santos CAG, et al. Geo-ecological impact assessment of severe cyclonic storm Amphan on Sundarban mangrove forest using geospatial technology. Estuar Coast Shelf Sci. 2021;260:107486. doi:10.1016/j.ecss.2021.107486.

[r23] Kumar R, Rani S, Maharana P. Assessing the impacts of Amphan cyclone over West Bengal, India: A multi-sensor approach. Environ Monit Assess. 2021;193(5):283. doi:10.1007/s10661-021-09124-9. 33871678

[r24] Sil S, Gangopadhyay A, Gawarkiewicz G, et al. Shifting seasonality of cyclones and western boundary current interactions in Bay of Bengal as observed during Amphan and Fani. Sci Rep. 2021;11(1):22052. doi:10.1038/s41598-021-01607-6. 34764378 PMC8586239

[r25] Paul S, Mishra M, Pati S, et al. Evaluation of overwash vulnerability and shoreline dynamics in cyclone-prone Sagar Island, Sundarbans (India). Sci Total Environ. 2024;907:167933. doi:10.1016/j.scitotenv.2023.167933. 37898194

[r26] Bhargava R, Friess DA. Previous shoreline dynamics determine future susceptibility to cyclone impact in the sundarban mangrove forest. Front Mar Sci. 2022;9:814577. doi:10.3389/fmars.2022.814577.

[r27] Pramanik M, Szabo S, Pal I, et al. Population health risks in multi-hazard environments: Action needed in the Cyclone Amphan and COVID-19 – hit Sundarbans region, India. Clim Dev. 2022;14(2):99–104. doi:10.1080/17565529.2021.1889948.

[r28] Khatoon S, Bhattacharya P, Mukherjee N, et al. Epidemic dynamics post-cyclone and tidal surge events in the bay of Bengal region. Ann Glob Health. 2025;91(1):39. 40718109 10.5334/aogh.4751 PMC12292050

[r29] UC Center for Climate, Health and Equity Program. Perspectives: COP29’s bold steps and shortcomings in the climate crisis fight. Published December 10, 2024. Accessed December 25, 2024. https://health.universityofcalifornia.edu/news/perspectives-cop29s-bold-steps-and-shortcomings-climate-crisis-fight.

[r30] Bhattacharya A, Songwe V, Soubeyran E, Stern N. Raising Ambition and Accelerating Delivery of Climate Finance. London School of Economics (LSE); 2024. https://www.lse.ac.uk/granthaminstitute/wp-content/uploads/2024/11/Raising-ambition-and-accelerating-delivery-of-climate-finance_Third-IHLEG-report.pdf.

[r31] United Nations Climate Change. The Baku to Belém Roadmap to 1.3T discussion paper: Building an effective diplomatic strategy. Published 2025. Accessed July 13, 2025. https://unfccc.int/sites/default/files/resource/C2ES_Baku_to_Belem_Roadmap_Building_an_effective_diplomatic_strategy.pdf.

[r32] Majumder S. Livelihood & socio-economic study on Sundarban area: A brief research review. IOSR J Econ Finance. 2024;16(1):23–29.

[r33] Roy A, De A, Aftabuddin M. Analysis of health ailments and associated risk factors in small-scale fisherfolk community of Indian Sundarbans: A cross-sectional study. Indian J Community Med. 2024;49(2):360–366. 38665455 10.4103/ijcm.ijcm_906_22 PMC11042129

[r34] Government of West Bengal. District Human Development Report: South 24 Parganas. Published 2009. Accessed 2025. https://www.undp.org/sites/g/files/zskgke326/files/migration/in/hdr_south24_parganas_2009_full_report.pdf.

[r35] Ghosh S, Mistri B. Cyclone-induced coastal vulnerability, livelihood challenges and mitigation measures of Matla-Bidya inter-estuarine area, Indian Sundarban. Nat Hazards (Dordr). 2023;116(3):3857–3878. 36817633 10.1007/s11069-023-05840-2 PMC9925922

[r36] Corbin JM, Strauss AC. Basics of Qualitative Research. 3rd ed. SAGE Publications; 2008.

[r37] Braun V, Clarke V. Using thematic analysis in psychology. Qual Res Psychol. 2006;3(2):77–101.

[r38] Ministry of Health and Family Welfare, Government of India. About Accredited Social Health Activist (ASHA). Accessed October 18, 2025. https://nhm.gov.in/index1.php?lang=1&level=1&sublinkid=150&lid=226.

[r39] Press Information Bureau. Schemes for Anganwadi workers. Published February 11, 2022. Accessed October 18, 2025. https://www.pib.gov.in/PressReleaseIframePage.aspx?PRID=1797679.

[r40] District Rural Development Cell (Anandadhara). Government of West Bengal. Published 2002. Accessed October 18, 2025. https://malda.gov.in/district-rural-development-cell-anandadhara/.

[r41] Kiger ME, Varpio L. Thematic analysis of qualitative data: AMEE Guide No. 131. Med Teach. 2020;42(8):846–854. 32356468 10.1080/0142159X.2020.1755030

[r42] Finlay L. Negotiating the swamp: The opportunity and challenge of reflexivity in research practice. Qual Res. 2002;2(2):209–230.

[r43] Census India. Kultali Block population, religion, caste South Twenty Four Parganas district, West Bengal. Published 2011. Accessed October 28, 2025. https://www.sundarbanaffairswb.in/home/page/kultali.

[r44] Census India. Gosaba Block population, religion, caste South Twenty Four Parganas district, West Bengal. Published 2011. Accessed October 28, 2025. https://www.sundarbanaffairswb.in/home/page/gosaba.

[r45] Lester D, Hvezda J, Sullivan S, et al. Maslow’s hierarchy of needs and psychological health. J Gen Psychol. 1983;109(1):83–85. 28150561 10.1080/00221309.1983.9711513

[r46] Chowdhury AN, Brahma A, Banerjee S, et al. Deliberate self-harm prevention in the Sundarbans region need immediate public health attention. J Indian Med Assoc. 2009;107(2):88–93. 19585816

[r47] Rahman K-S, Dana NH, Rahman MM, et al. Degradation of mangrove forests in the Sundarbans: An assessment based on perspectives of mangrove resource collectors using the DPSIR framework. Trees For People. 2025;19:100769.

[r48] Farmer PE, Nizeye B, Stulac S, et al. Structural violence and clinical medicine. PLoS Med. 2006;3(10):e449. 17076568 10.1371/journal.pmed.0030449 PMC1621099

[r49] Tong A, Sainsbury P, Craig J. Consolidated criteria for reporting qualitative research (COREQ): A 32-item checklist for interviews and focus groups. Int J Qual Health Care. 2007;19(6):349–357. 17872937 10.1093/intqhc/mzm042

[r50] UN Trade and Development (UNCTAD). Countries agree $300 billion by 2035 for new climate finance goal – what next? Published 2024. Accessed June 16, 2025. https://unctad.org/news/countries-agree-300-billion-2035-new-climate-finance-goal-what-next.

[r51] United Nations Climate Change. Setting the scene for COP30 – from promise to practice. Published March 25, 2025. Accessed July 13, 2025. https://unfccc.int/news/setting-the-scene-for-cop30-from-promise-to-practice.

[r52] Schlanger Z. Climate diplomacy’s $300 billion failure. The Atlantic. Published November 24, 2024. Accessed December 25, 2024. https://www.theatlantic.com/science/archive/2024/11/cop-climate-baku-outcome-finance/680789/.

