# Supplementary figures and images for: Frontlines of Climate Change and Global Health Inequity: How Recurring Cyclones Undermine Health, Livelihoods, and Development in the Indian Sundarbans
Source: Ann Glob Health. 2026 Apr 1;92(1):28. doi: 10.5334/aogh.5074 (PMC13062752; doi:10.5334/aogh.5074)

Figure 1.

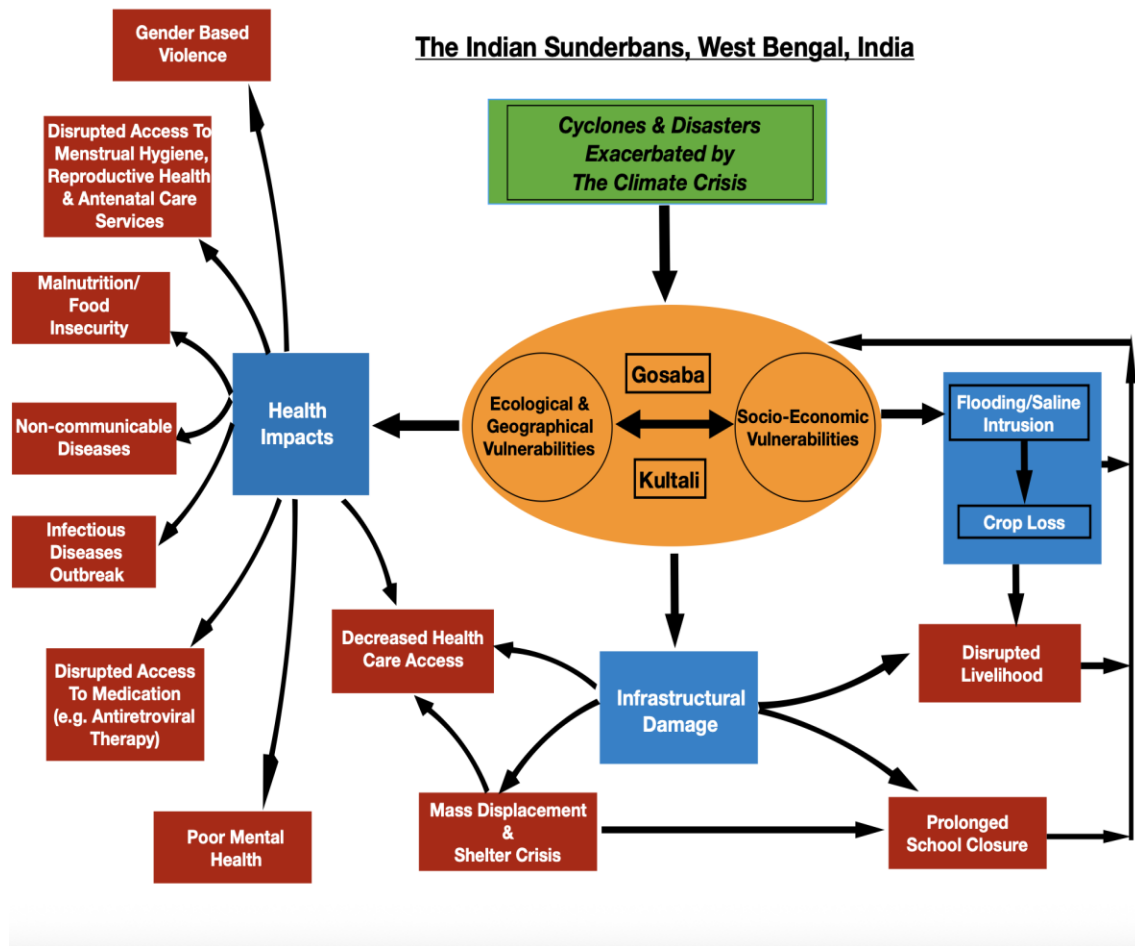

Supplement: Supplementary Material. — Figure 1. [file agh-92-1-5074-s1.pdf]
