# Supplementary material for: Frontlines of Climate Change and Global Health Inequity: How Recurring Cyclones Undermine Health, Livelihoods, and Development in the Indian Sundarbans
Source: Ann Glob Health. 2026 Apr 1;92(1):28. doi: 10.5334/aogh.5074 (PMC13062752; doi:10.5334/aogh.5074)
Supplement: Supplementary Appendix A. — Semi-Structured Interview Guide. [file agh-92-1-5074-s2.pdf]

Appendix A:

Semi-Structured Interview Guide

Introduction:

Hello, my name is _______ and I'm a _______ from ______. I'm conducting a qualitative research study exploring how climate-related events –such as Cyclone Amphan, Cyclone Dana, and heat stress—have impacted communities in various regions of West Bengal.

We're particularly interested in understanding the long-term effects of these events on public health, mental well-being, displacement, and local economic challenges. This interview is part of a larger study in collaboration with the Child in Need Institute (CINI), All India Institute of Hygiene & Public Health, and Division of Global Health Equity at Brigham and Women's Hospital.

Thank you so much for being willing to meet and share your perspective. The information you share today will remain anonymous and be used only for academic and public health research purposes.

At this point, I will confirm that the participant submitted their written or verbal consent form.

Interview Information

Name of Interviewer	
Date of Interview (mm/dd/yyyy)	
Total Duration of Interview (Hour(s) / Minute(s))	
Data Recording (Audio / Handwritten Notes / Other)	
Verbal Consent to Participate (Yes / No)	
Verbal Consent to Audio Record (Yes / No)	
Any Questions Before We Begin the Interview?	

Let me know when you're ready to begin.

First, I would like to begin by collecting some demographic information.

1. Demographic Information

Age	
Gender Identity	
Professional Role (e.g., NGO leader, public health official, local gov't official)	
Years of Experience in Current Role	
Years of Experience Working in the Sundarbans	

2. Interview Questions

Background

Could you please tell me about your work?

How long have you been working in this region?

Awareness, Observations, and Short-Term/Long-Term Impacts of Cyclones

Which major climate-related events have occurred during your time living and working in this region?

Could you please describe your personal/professional experiences responding to/navigating impacts of cyclones and flooding events?

Can you describe the immediate and long-term impacts these events have had on local communities?

How was health and healthcare delivery impacted?

How was access to food impacted?

How did these disasters affect housing/shelter?

How did these disasters affect the economy/employment?

Did these disasters result in the displacement of families?

Did these disasters impact children's attendance in schools?

Have children had to drop out of school to support their families? If so, what kinds of work do they do?

How might these impacts intersect with issues of gender equity?

How did these disasters affect women and women's health?

What are the biggest economic challenges people have faced after these disasters?

What support systems exist to assist families in economic recovery?

Describe the community's understanding of climate change – is something people are often worrying about?

Displacement

Have you or your family ever had to leave your home because of flooding, erosion, or other environmental reasons?

How did that experience affect you mentally and emotionally?

Do you worry about needing to move again in the future?

Mental Health

How do you feel when you think about the changes in weather or disasters like cyclones and floods?

After a natural disaster, how do you usually cope emotionally?

Do you often feel anxious, fearful, or stressed about the future because of changes in the climate?

Have you or your family members experienced trouble sleeping, loss of appetite, or sadness due to climate-related events?

Have you ever spoken to a health worker/health professional (doctor, nurse, ASHA worker ect.) about feeling anxious or stressed?

Are there services in your village or nearby where you can go for mental health support?

What kinds of traditional practices or local support do you use to cope with emotional stress?

Have you observed any mental health concerns among community members post-disaster?

What types of symptoms or challenges have you witnessed (e.g., anxiety, trauma, isolation)?

What mental health support or services are available locally, if any?

Social and Community Support

Who do you turn to when you are feeling overwhelmed or stressed?

Are there people in your community who support each other during or after natural disasters?

Do you feel that your community has become more or less close due to the challenges brought by climate change? Or has it triggered the competition for resources?

Hopes and Fears for the Future

What are your biggest worries for the future related to the environment?

How do you think the younger generation in your community is affected by the changes in nature?

What kind of help or support would make you feel more prepared or secure?

Strategies & Solutions

What are strengths in the community that help the community respond to these cyclone events?

What community organizations are supporting families in response to cyclones in coastal India?

What services do they offer?

In what ways is the government providing adequate responses to these cyclones?

What are gaps that should be addressed?

Needs and Recommendations

What types of interventions do you suggest? Counseling? Government-Led Recovery Efforts (technology, skill development, alternative livelihood, climate resilient infrastructure, early warning systems)

If a well-paying job was presented to your community focused on reforestation that would protect the community from these harms, is that something you would be interested in? If so, why or why not?

What does the community need to respond appropriately to severe cyclones?

What are specific recommendations you would advise to (mental health, economic distress, migration, educational opportunities gender equity)

What kinds of support (e.g., mental health, education, livelihood programs) would be most helpful?

If you could make one policy or programmatic recommendation, what would it be?

Conclusion

Is there anything else you would like to say or add to our conversation?

Finally, I just want to reaffirm that I have your consent to include your responses and feedback in the data analysis of this investigation. Could you please reaffirm your consent?

Thank you so much for your participation!
